# 固相萃取-高效液相色谱-串联质谱法同时测定环境水样中22种抗生素

**DOI:** 10.3724/SP.J.1123.2022.06004

**Published:** 2023-03-08

**Authors:** Jin WANG, Kaixiao YE, Yan TIAN, Ke LIU, Liuling LIANG, Qingqian LI, Ning HUANG, Xinting WANG

**Affiliations:** 1.广西壮族自治区生态环境监测中心, 广西 南宁 530028; 1. Guangxi Zhuang Autonomous Region Ecological and Environmental Monitoring Center, Nanning 530028, China; 2.广西大学资源环境与材料学院, 广西 南宁 530004; 2. School of Resources, Environment and Materials, Guangxi University, Nanning 530004, China

**Keywords:** 高效液相色谱-串联质谱, 固相萃取, 抗生素, 环境水样, high performance liquid chromatography-tandem mass spectrometry (HPLC-MS/MS), solid phase extraction (SPE), liquid, antibiotics, environmental water samples

## Abstract

抗生素作为环境介质中一种典型的新污染物,在各类环境水样中检出频率高且浓度低。为快速、灵敏、准确地分析各类水体中的抗生素,建立了固相萃取-高效液相色谱-串联质谱法(SPE-HPLC-MS/MS)同时测定环境水样中4种青霉素类、12种喹诺酮类和6种大环内酯类共22种抗生素的分析方法。针对抗生素特性和样品基质特点优化前处理方法,重点优化固相萃取柱、水样pH值、水样中乙二胺四乙酸二钠(Na_2_EDTA)加入量。在200 mL水样中加入0.5 g Na_2_EDTA,并调节水样的pH值至3,经HLB固相萃取柱富集净化,以乙腈-0.15%(v/v)甲酸水溶液为流动相进行梯度洗脱,采用电喷雾电离源,在正离子模式下使用多反应监测模式进行定性定量分析。结果显示,22种抗生素的相关系数(*r*)≥0.995,呈现良好的线性关系,方法检出限和定量限分别为2.3~10.7 ng/L和9.2~42.8 ng/L,地表水中3个水平下的加标回收率为61.2%~157%,相对标准偏差(RSD)为1.0%~21.9%,废水中3个水平下的加标回收率为50.1%~129%, RSD为1.2%~16.9%。该方法成功用于水库、地表水、污水处理厂出口、畜禽养殖场等不同类型水样中抗生素的同时测定,其中地表水和畜禽养殖废水中大部分抗生素有检出,在地表水中林可霉素检出率为90%,在畜禽养殖废水中氧氟沙星的检出含量最高,为127 ng/L。该方法检出限和回收率满足定量分析要求,且具有富集水样体积少、分析时间短、适用范围广等优势,特别适用于突发环境污染应急监测,同时为摸清新污染物环境赋存底数和新污染物治理与管控提供有力支撑。

## Abstract (en)

The widespread and frequent use of antibiotics to treat diseases or encourage animal growth has resulted in their persistence and accumulation in water, soil, and sediments. As a typical emerging pollutant in the environment, antibiotics have become an important research focus in recent years. Antibiotics are commonly found at trace levels in water environments. Unfortunately, the determination of various types of antibiotics, all of which exhibit different physicochemical properties, remains a challenging endeavor. Thus, developing pretreatment and analytical techniques to achieve the rapid, sensitive, and accurate analysis of these emerging contaminants in various water samples is an essential undertaking.In this paper, a solid phase extraction-high performance liquid chromatography-tandem mass spectrometry (SPE-HPLC-MS/MS) method for the simultaneous determination of 22 antibiotics including 4 penicillins, 12 quinolones and 6 macrolides in environmental water samples was developed. Based on the characteristics of the screened antibiotics and sample matrix, the pretreatment method was optimized, focusing on the SPE column, pH of the water sample, and amount of ethylene diamine tetra-acetic acid disodium (Na_2_EDTA) added to the water sample. Prior to extraction, a 200 mL water sample was added with 0.5 g of Na_2_EDTA and pH-adjusted to 3 using sulfuric acid or sodium hydroxide solution. Water sample enrichment and purification were achieved using an HLB column. HPLC separation was carried out on a C_18_ column (100 mm×2.1 mm, 3.5 μm) via gradient elution with a mobile phase composed of acetonitrile and 0.15% (v/v) formic acid aqueous solution. Qualitative and quantitative analyses were performed on a triple quadrupole mass spectrometer in multiple reaction monitoring mode using an electrospray ionization source. The results showed correlation coefficients greater than 0.995, indicating good linear relationships. The method detection limits (MDLs) and limits of quantification (LOQs) were in the ranges of 2.3-10.7 ng/L and 9.2-42.8 ng/L, respectively. The recoveries of target compounds in surface water at three spiked levels ranged from 61.2% to 157%, with relative standard deviations (RSDs) of 1.0%-21.9%. The recoveries of target compounds in wastewater at three spiked levels were 50.1%-129%, with RSDs of 1.2%-16.9%. The method was successfully applied to the simultaneous determination of antibiotics in reservoir water, surface water, sewage treatment plant outfall, and livestock wastewater. Most of the antibiotics were detected in watershed and livestock wastewater. Lincomycin was detected in 10 surface water samples, with a detection frequency of 90%, and ofloxacin showed the highest contents (127 ng/L) in livestock wastewater. Therefore, the present method exhibits excellent performance in terms of MDLs and recoveries compared with previously reported methods. The developed method presents the advantages of small water sample volumes, wide applicability, and fast analysis times; thus, it can be considered a rapid, efficient, and sensitive analytical method with excellent potential for monitoring emergency environmental pollution. The method could also provide a reliable reference for formulating antibiotic residue standards. The results provide strong support for and an improved understanding of the environmental occurrence, treatment, and control of emerging pollutants.

抗生素主要用于人类和动物疾病的预防和治疗,且作为生长促进剂广泛用于农业和畜禽养殖业中^[1]^。研究显示,2010~2020年全球抗生素使用量增加了74.5%^[2]^,世界各国对抗生素的依赖性持续增加,预计到2030年,抗生素的使用量将增加2倍以上^[3]^。全球抗生素年使用量为10~20万吨,中国每年使用超过2.5万吨,是世界上生产和使用抗生素最多的国家^[4,5]^。由于大部分的抗生素在人类和动物体内不能完全被消化^[6]^,大约有5%~90%的抗生素会以其代谢物或者母体化合物的形式排放到水环境中^[7]^。近年来,已经在世界范围内的水环境中检测出抗生素残留^[8,9]^,这可能会对生态系统甚至人类健康产生潜在的威胁^[10]^。

抗生素作为一种新污染物,在环境领域得到了越来越多的关注^[11]^。2022年国家首次将新污染物调查试点监测纳入生态环境监测体系。今年国务院印发了《新污染物治理行动方案》,方案中将规范抗生素药物的使用作为工作目标之一,并将抗生素纳入了《重点管控新污染物清单》,列为四大典型新污染物之一。近年来,已经在世界范围内的水环境中检测出抗生素残留,我国七大水系(松花江、大辽河、海河、黄河、淮河、长江、珠江)水体中均检出*β*-内酰胺类、氟喹诺酮类、大环内酯类氯霉素、磺胺类和四环素类等6大类抗生素,含量分布为ng/L~μg/L^[12⇓-14]^;马健生等^[15]^报道了在哈尔滨市地下水中检出6大类抗生素,以磺胺类、喹诺酮、大环内酯类和四环素为主;丁紫荣等^[16]^在某水产养殖的养殖废水中检出9种氟喹诺酮类抗生素。采用固相萃取技术富集净化样品,液相色谱-串联质谱法分析检测抗生素是国内外研究员最常用的手段。然而,抗生素多种多样,性质差异大,在环境介质中浓度低,文献报道的方法大多存在检测的抗生素种类少、耗时较长、灵敏度低和适用范围单一等问题^[16⇓-18]^,难以实现同时高灵敏快速分析检测不同环境水体中多种类抗生素。

结合我国抗生素的使用情况和文献报道抗生素的检出情况,确定常用和常检出的3类(包括4种青霉素类、12种喹诺酮类和6种大环内酯类)共22种抗生素为目标化合物,采用固相萃取-高效液相色谱-串联质谱技术(SPE-HPLC-MS/MS),针对抗生素特性和样品基质特点优化前处理方法、色谱条件和质谱条件,建立同时测定环境水样中22种抗生素的分析方法。通过测定检出限和基质加标回收率评价方法的灵敏度、准确度和方法重复性,证明方法的可靠性。方法成功应用于水库、地表水、污水处理厂出口和畜禽养殖场等不同类型水样中抗生素的分析检测。本方法富集水样体积少,仪器分析时间短,可有效缩短样品分析时间,达到快速检测的目的,且适用于不同水样,为抗生素残留的标准制定和实际环境监测尤其突发环境污染应急监测提供补充和参考,同时为摸清新污染物环境赋存底数和新污染物治理与管控提供有力支撑。

## 1 实验部分

### 1.1 仪器、试剂与材料

1260-6460高效液相色谱-三重四极杆质谱仪(美国Agilent公司); Aqua Trace ASPE 799全自动固相萃取仪(日本GL Science公司); Milli-Q超纯水仪(美国Millipore公司); Turbo Vap Ⅱ氮吹浓缩仪(美国Biotage公司); Oasis HLB萃取小柱(美国Waters公司)。

甲醇和乙腈均为色谱纯,购自美国Fisher公司;甲酸为色谱纯,购自上海安谱实验科技股份有限公司;乙二胺四乙酸二钠(Na_2_EDTA)和氢氧化钠均为优级纯,购自天津市光复科技发展有限公司;硫酸为优级纯,购自成都市科隆化学品有限公司。

林可霉素(lincomycin,纯度98.2%)、青霉素G(penicillin G,纯度99.0%)、麻保沙星(marbofloxacin,纯度99.0%)、氟罗沙星(fleroxacin,纯度98.0%)、氧氟沙星(ofloxacin,纯度99.0%)、诺氟沙星(norfloxacin,纯度99.0%)、环丙沙星(ciprofloxacin,纯度94.0%)、恩诺沙星(enrofloxacin,纯度99.0%)、奥比沙星(orbifloxacin,纯度97.8%)、沙氟沙星(sarafloxacin,纯度97.0%)、双氟哌酸(difloxacin,纯度98.0%)、司帕沙星(sparfloxacin,纯度98.9%)、克林霉素(clindamycin,纯度98.0%)、竹桃霉素(oleandomycin,纯度98.8%)、泰乐霉素(tylosin,纯度99.0%)、青霉素V(penicillin V,纯度98.8%)、吉他霉素(leucomycin A5,纯度92.0%)、恶喹酸(oxolinic acid,纯度98.0%)、氟甲喹(flumequine,纯度98.3%)、氯唑西林(cloxacillin,纯度95.0%)、交沙霉素(josamycin,纯度99.0%)和双氯西林(dicloxacillin,纯度97.5%)均购自德国Dr. Ehrenstorfer公司。

分别称取适量抗生素标准品,氟罗沙星和诺氟沙星用2%甲酸甲醇溶液溶解,其余抗生素均用甲醇溶解,配制成质量浓度为1.0 mg/mL的单一标准储备液,并用甲醇配制成1.0 mg/L的22种抗生素混合标准储备液,于-20 ℃冰箱储存。使用时,用甲醇稀释至所需浓度。

### 1.2 样品前处理

量取200 mL水样,加入0.5 g Na_2_EDTA,使用硫酸或氢氧化钠溶液调节水样的pH值至3,水样以5 mL/min的流速通过预先活化的Oasis HLB固相萃取柱。HLB柱依次用10 mL甲醇和10 mL超纯水进行活化,在活化过程中确保小柱中填料表面不露出液面。样品富集后,用10 mL超纯水淋洗小柱,去除杂质。用氮气吹干小柱30 min。先用3 mL 0.1%甲酸甲醇溶液浸泡小柱5 min,再用9 mL 0.1%甲酸甲醇溶液以1 mL/min的速度洗脱样品,收集全部洗脱液。将洗脱液氮吹浓缩至1.0 mL以下,用0.1%甲酸甲醇定容至1.0 mL,涡旋混匀后,过0.22 μm滤膜,滤液置于进样瓶中,待测。

### 1.3 分析条件

色谱柱:ZORBAX SB-C_18_色谱柱(100 mm×2.1 mm, 3.5 μm);柱温:35 ℃;流速:0.5 mL/min;进样体积:5.0 μL;流动相:A为0.15%(v/v)甲酸水溶液,B为乙腈。梯度洗脱程序:0~7.0 min, 10%B~25%B; 7.0~7.1 min, 25%B~65%B; 7.1~12.0 min, 65%B~80%B; 12.0~12.1 min, 80%B~10%B; 12.1~14.0 min, 10%B。

离子源:电喷雾电离(ESI)源,采用正离子模式检测。毛细管电压:4.0 kV。干燥气和雾化气均为氮气(N_2_),干燥气温度为350 ℃,干燥气流速为11 L/min,雾化气压力为0.17 MPa。采用多反应离子监测(MRM)模式,目标化合物的质谱参数见表1。

**表1:** 目标化合物的质谱参数

Compound	Precursor ion (m/z)	Product ions (m/z)	Fragm- entor/V	Collision energies/eV
Lincomycin	407.0	359.0, 126.0^*^	140	30, 15
Penicillin G	335.0	289.0, 91.0^*^	160	23, 50
Marbofloxacin	363.0	344.9, 72.1^*^	120	20, 25
Fleroxacin	369.9	326.0^*^, 269.0	140	15, 25
Ofloxacin	361.9	317.9^*^, 260.8	140	15, 25
Norfloxacin	320.0	302.0, 276.0^*^	120	20, 15
Ciprofloxacin	332.0	313.9, 288.0^*^	130	20, 15
Enrofloxacin	360.0	341.9, 316.0^*^	130	20, 15
Orbifloxacin	395.9	378.0, 352.0^*^	140	15, 15
Sarafloxacin	386.0	367.8, 342.0^*^	140	20, 15
Difloxacin	400.0	381.9, 356.0^*^	140	20, 15
Sparfloxacin	393.0	349.0^*^, 291.8	150	15, 25
Clindamycin	425.0	377.0, 126.0^*^	140	30, 15
Oleandomycin	688.3	544.2, 158.0^*^	150	30, 15
Tylosin	916.8	772.8, 174.3^*^	230	40, 30
Penicillin V	351.0	256.8, 228.9^*^	170	8, 10
Leucomycin A5	772.7	173.9^*^, 109.2	200	30, 40
Oxolinic acid	261.9	243.9^*^, 216.1	100	15, 30
Flumequine	261.9	243.9^*^, 202.1	110	15, 35
Cloxacillin	435.9	220.1, 177.9^*^	190	15, 25
Josamycin	828.8	174.1^*^, 109.1	210	35, 50
Dicloxacillin	470.0	451.9, 211.8^*^	190	15, 25

* Quantitative ion.

## 2 结果与讨论

### 2.1 液相色谱-串联质谱条件优化

#### 2.1.1 液相色谱条件优化

目标化合物中青霉素类、大环内酯类和喹诺酮类抗生素的分子结构分别包含*β*-内酰胺环、二甲氨基、羧基,这些特征基团使得抗生素具有一定的水溶性,不同的色谱柱对其分离效果影响较大。实验考察了不同填料、不同粒径和不同长度的色谱柱对目标物的分离效果,即使用ZORBAX Eclipse Plus C_18_(100 mm×4.6 mm, 3.5 μm)、ZORBAX SB-C_18_(100 mm×2.1 mm, 3.5 μm)和ZORBAX SB-C_18_(50 mm×2.1 mm, 1.8 μm)色谱柱,优化柱温、流速和梯度洗脱程序,结果显示在各自优化的色谱条件下,使用Plus C_18_柱时,青霉素V、氯唑西林、双氯西林、交沙霉素、沙氟沙星和双氟沙星等目标物不出峰,泰乐霉素和吉他霉素色谱峰拖尾,其他喹诺酮抗生素完全重叠未能分离;使用SB-C_18_短柱时,泰乐霉素、吉他霉素和喹诺酮类抗生素色谱峰展宽严重;使用SB-C_18_长柱时,峰形对称,而且响应强度相对较高。最终选择ZORBAX SB-C_18_色谱柱(100 mm×2.1 mm, 3.5 μm)。

为使抗生素获得更好的分离效果、色谱峰形和灵敏度,以提高分析的准确度,进一步优化了流动相。甲酸可以增加氢离子浓度,促进[M+H]^+^分子离子峰的形成,提高离子化效率和灵敏度,同时也会抑制离子化效率^[19]^。故实验考察了水相中加入不同体积分数的甲酸(0.05%、0.1%、0.15%、0.2%、0.25%)对目标化合物的色谱峰形和灵敏度的影响,如图1所示。结果表明:水相中未添加甲酸时,只有恶喹酸和氟甲喹出峰,且峰展宽,拖尾严重,其他组分不出峰,这是因为水相中未能提供较强的质子;随着水相中甲酸体积分数的增加,各组分的色谱峰形越来越窄,柱容量大,色谱峰形对称,特别是甲酸体积分数从0.15%开始,色谱峰形明显改善,尤其是喹诺酮类抗生素色谱峰拖尾和峰展宽的问题得到了显著改善;麻保沙星、氟罗沙星、氧氟沙星、诺氟沙星、环丙沙星、恶喹酸和氟甲喹喹诺酮类抗生素的响应强度随着甲酸体积分数的增大而增强,但当甲酸体积分数为0.25%时开始下降。

**图1:**
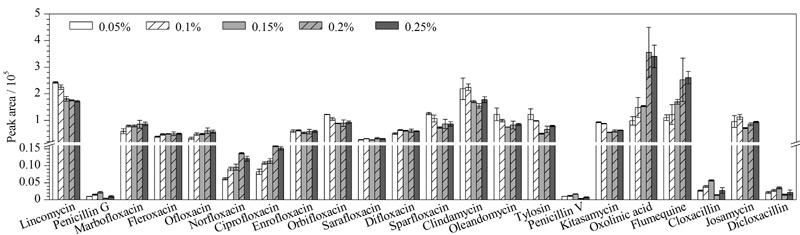
流动相中甲酸体积分数对目标化合物峰面积的影响(*n*=3)

恩诺沙星、沙氟沙星和双氟沙星的响应强度无明显变化。大环内酯类抗生素林可霉素、克林霉素、竹桃霉素、泰乐霉素和吉他霉素的响应强度随着甲酸体积分数的增大明显减弱。青霉素V、青霉素G、氯唑西林和双氯西林在甲酸体积分数为0.2%时,响应较低。

综合峰形和灵敏度的考虑,最终选择0.15%甲酸水溶液作为水相,乙腈作为有机相,色谱图见图2。

**图2:**
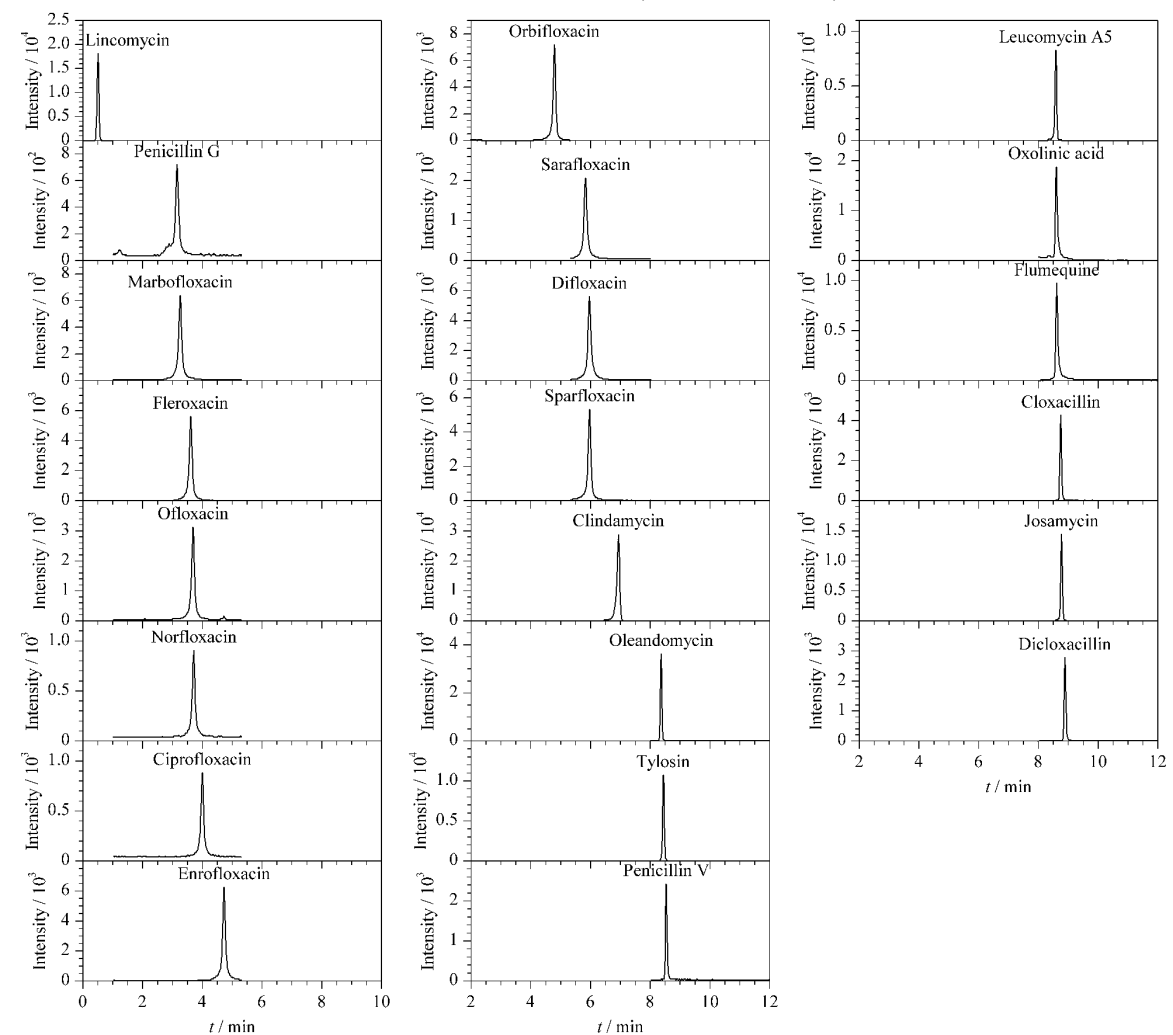
22种抗生素标准溶液(50.0 μg/L)的色谱图

#### 2.1.2 质谱条件优化

配制质量浓度为1.0 mg/L的单一抗生素标准溶液,采用正离子模式逐一对各个抗生素进行一级质谱母离子全扫描,22种抗生素的准分子离子峰均为[M+H]^+^。然后优化碎裂电压,再进行二级质谱子离子扫描,对母离子进行碎裂得到碎片离子,选择响应高、稳定和质量数大的碎片离子为定量离子,响应较低的为定性离子,最后形成2个离子对并采用MRM模式优化碰撞能,最终得到22种抗生素的质谱参数(见表1)。

### 2.2 样品前处理条件优化

#### 2.2.1 固相萃取柱的选择

环境水样基质复杂,抗生素在水样中浓度低,常采用固相萃取法进行富集和净化,不同填料的固相萃取柱对抗生素具有不同的萃取效率,实验采用C_18_柱和HLB柱进行加标回收试验,每组6个样品,以对比C_18_柱和HLB柱对目标化合物的萃取效果,结果如图3a所示,喹诺酮类抗生素在C_18_柱上的萃取效果比较差,其平均回收率均低于50%,所有目标物在HLB柱上的萃取效果均比较理想,回收率明显高于C_18_柱。可能是由于目标物为中等极性和极性化合物,根据“相似相溶”原理,C_18_柱填料为亲酯性的,适用于中等极性和非极性化合物,HLB柱的填料为亲水亲酯性聚合物,能很好地保留亲水性物质,对抗生素具有较好的萃取效果。崔敬鑫等^[18]^在萃取环境水样中喹诺酮类、磺胺类、大环内酯类和四环素类抗生素时也发现HLB柱萃取效果优于C_18_柱。因此,本实验选择HLB柱萃取水样中抗生素。

**图3:**
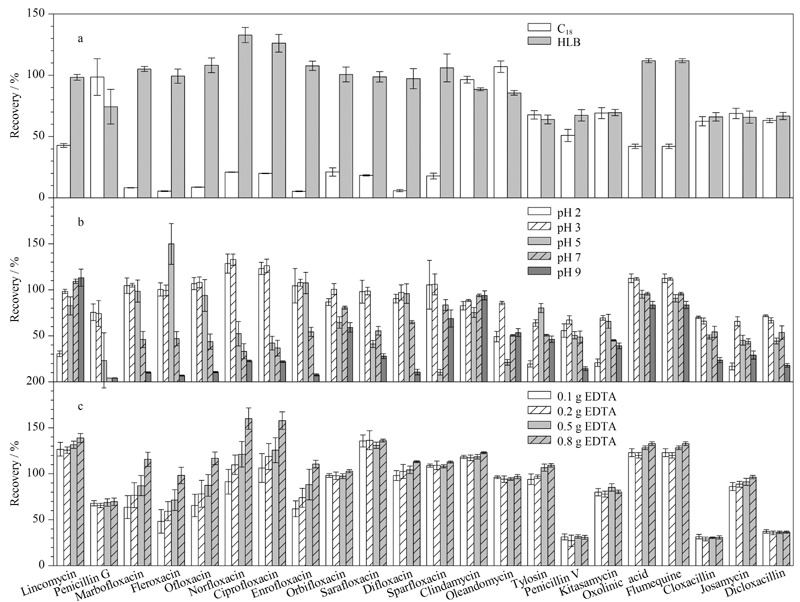
(a)固相萃取柱、(b)水样pH值和(c)水样中Na_2_EDTA加入量对目标化合物回收率的影响(*n*=6)

#### 2.2.2 水样pH值的优化

抗生素在固相萃取柱上的萃取效果与抗生素的酸度系数(p*K*_a_)和水样的pH值有关,即水样pH值会改变抗生素在水中存在形式而影响萃取效率^[19]^。本实验研究的抗生素理化性质差异较大,青霉素类为弱酸性化合物,大环内酯类为碱性化合物,喹诺酮类为两性化合物,在选用pH适用范围广、通用性强的HLB柱前提下优化水样的pH值尤为重要。实验将水样pH值调至2、3、5、7、9进行加标回收试验,每组6个样品,结果如图3b所示,与pH值为3相比较,pH值为2时,大环内酯类抗生素平均回收率下降,青霉素类和喹诺酮类抗生素的平均回收率无显著差异;pH值为5时,青霉素G、环丙沙星、沙氟沙星、司帕沙星、竹桃霉素等抗生素平均回收率小于50%;大部分目标化合物在中性和碱性条件下平均回收率明显下降。为保证目标物均可获得较好的回收率,选取水样pH值为3进行富集。

#### 2.2.3 水样中Na_2_EDTA加入量的优化

复杂水样中含有较多的多价态金属离子和腐殖酸,而金属离子可与喹诺酮类、大环内酯类等抗生素发生反应形成络合物从而影响萃取效率。因此,常在水样中加入金属络合剂Na_2_EDTA,与金属离子络合,从而释放出抗生素,进一步有效提高抗生素的萃取效率^[20⇓-22]^。实验比较了在200 mL地表水中分别加入0.1、0.2、0.5、0.8 g Na_2_EDTA对目标化合物回收率的影响。结果如图3c所示,随着Na_2_EDTA加入量的增加,青霉素类抗生素的萃取回收率无明显变化,而其他类抗生素的萃取回收率显著提升。但是,当Na_2_EDTA加入量为0.8 g时,诺氟沙星、环丙沙星、沙氟沙星、氟甲喹和恶喹酸等喹诺酮类抗生素回收率超过130%,可能是受到基体效应增强的影响,尤其当基体含有较多腐殖酸时^[23]^。综合考虑,本实验选择在水样中加入0.5 g Na_2_EDTA。

### 2.3 基质效应考察

分别采用空白水样的前处理液和甲醇配制一系列不同质量浓度的基质标准溶液和溶剂标准溶液,按优化好的液相色谱条件和质谱参数进行分析,以目标化合物质量浓度为横坐标,定量离子的峰面积为纵坐标,进行线性回归,绘制得到基质匹配标准曲线和溶剂标准曲线。基质效应(ME)=(基质匹配标准曲线斜率-溶剂标准曲线斜率)/溶剂标准曲线斜率×100%^[24]^,当|ME|<20%时,表示弱基质效应,当|ME|为20%~50%时,表示中等基质效应,当|ME|>50%时,表示强基质效应^[25]^。22种抗生素在水中的基质效应如图4所示。结果表明,22种抗生素在水中存在一定强度的基质增强或抑制效应,其中50%抗生素的|ME|<10%,无明显基质效应;31.8%抗生素的|ME|在10%~20%之间,表现出弱基质效应;其余4种抗生素的|ME|在20%~25%之间,表现出中等强度基质效应。大部分抗生素表现出弱基质效应,只有少部分抗生素表现出中等强度基质效应,且|ME|虽大于20%但未超出许多,综合考虑,实验采用溶剂标准曲线进行定量分析。

**图4:**
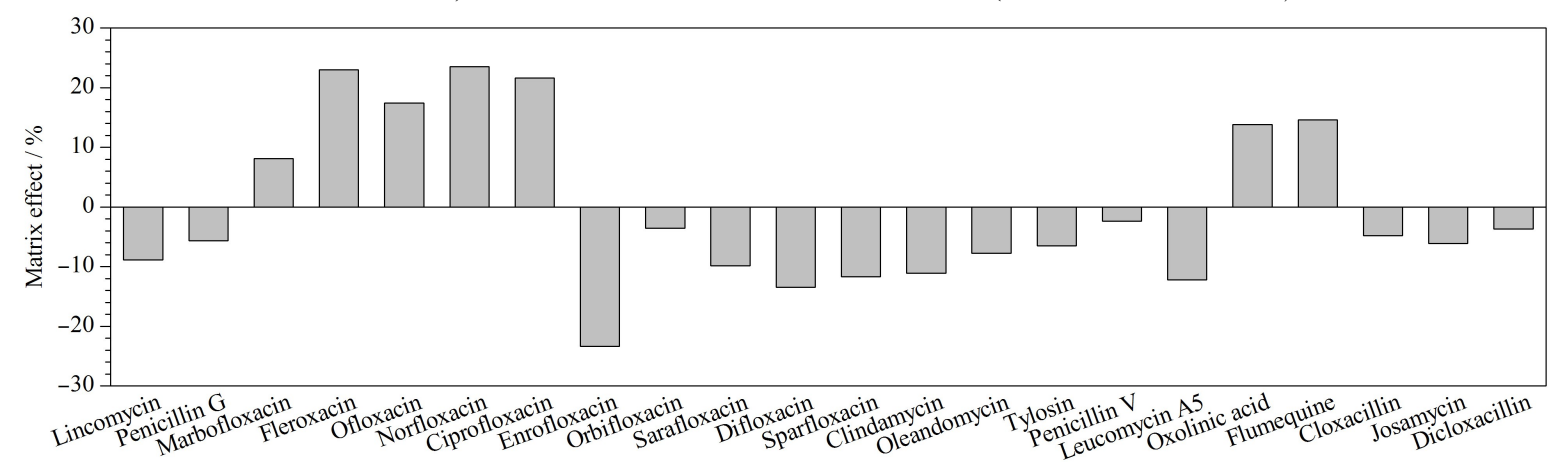
22种抗生素在水中的基质效应

### 2.4 方法性能

#### 2.4.1 线性关系、方法检出限和定量限

测定采用甲醇配制的一系列质量浓度标准溶液,以各目标化合物的峰面积为纵坐标,质量浓度为横坐标,考察线性关系。结果显示,目标化合物在相应的浓度范围内具有良好的线性关系,相关系数(*r*)≥0.995(见表2)。按照环境监测分析方法标准制修订技术导则^[26]^进行方法检出限(MDL)试验,即分别在7个200 mL空白水样中加入5.0 μL质量浓度为1 mg/L的混合标准溶液,按照1.2节和1.3节进行样品前处理和分析测定,计算7次测定结果的标准偏差,MDL为3.143倍的标准偏差,定量限(LOQ)为4倍的MDL。22种目标化合物的MDL为2.3~10.7 ng/L, LOQ为9.2~42.8 ng/L。

**表2:** 目标化合物的回归方程、线性范围、相关系数、方法检出限、定量限、加标回收率和精密度(*n*=6)

Compound	Regression equation	r	Linear range/ (μg/L)	MDL/ (ng/L)	LOQ/ (ng/L)	Surface water																Urban waste water															
						25 ng/L				100 ng/L						250 ng/L						25 ng/L						100 ng/L						250 ng/L			
						Re./ %	RSD/ %			Re./ %		RSD/ %				Re./ %		RSD/ %				Re./ %		RSD/ %				Re./ %		RSD/ %				Re./ %		RSD/ %	
Lincomycin	y=2229.45x-3812.90	0.998	2-160	8.1	32.4	120	5.1				122		3.8				127		2.8				113		2.7				121		2.1				120		2.8
Penicillin G	y=90.89x-153.76	0.999	2-160	9.3	37.2	111	8.3				114		2.3				102		5.2				128		12.2				126		2.1				115		3.4
Marbofloxacin	y=867.25x-3960.57	0.995	5-160	5.7	22.8	126	6.1				94.8		14.0				97.5		12.6				95.0		4.1				83.3		8.5				79.1		10.5
Fleroxacin	y=534.56x-2469.78	0.999	5-160	7.8	31.2	120	7.8				80.9		14.0				81.4		15.1				94.8		3.8				83.5		9.6				76.3		10.4
Ofloxacin	y=319.21x-1406.60	0.995	5-160	7.4	29.6	132	6.8				99.7		15.0				106		12.7				51.8		2.1				68.0		9.9				73.8		7.4
Norfloxacin	y=167.45x-641.00	0.998	5-100	10.7	42.8	116	11.4				94.1		20.1				92.7		21.9				50.1		1.9				73.2		11.1				66.6		10.1
Ciprofloxacin	y=166.99x-330.58	0.995	2-100	8.1	32.4	157	2.9				101		15.3				114		6.3				96.5		5.3				96.9		11.6				84.7		9.4
Enrofloxacin	y=799.09x-906.65	0.998	2-100	5.1	20.4	92.3	1.4				73.1		13.0				80.6		11.0				108		6.1				90.1		7.9				83.6		4.5
Orbifloxacin	y=1083.85x-1562.31	0.998	2-100	7.1	28.4	121	4.7				86.5		2.5				85.1		4.4				75.1		9.3				82.9		2.1				80.1		3.4
Sarafloxacin	y=369.11x-685.07	0.996	2-100	6.3	25.2	140	3.0				102		3.5				117		3.6				92.0		7.3				108		3.6				99.0		3.3
Difloxacin	y=893.39x-492.10	0.996	2-160	3.9	15.6	94.3	3.9				79.5		7.1				82.1		3.5				114		7.4				110		3.6				105		2.5
Sparfloxacin	y=723.65x-1003.24	0.997	2-100	3.8	15.2	127	4.3				94.6		1.9				96.4		4.9				62.2		10.6				82.9		2.4				79.5		2.6
Clindamycin	y=2872.32x-170.75	0.999	2-160	5.6	22.4	102	1.4				111		2.1				115		1.6				112		3.1				119		2.3				114		2.0
Oleandomycin	y=1781.20x+851.51	0.999	2-160	9.7	38.8	84.2	3.9				85.2		2.3				88.8		2.7				77.4		4.8				84.4		2.4				84.6		1.5
Tylosin	y=413.73x-26.90	0.999	2-160	2.4	9.6	91.6	2.6				81.2		1.5				82.8		2.5				78.6		2.9				87.4		1.8				83.2		2.8
Penicillin V	y=135.46x-97.73	0.999	2-160	6.1	24.4	66.5	11.0				86.7		5.1				82.0		3.7				116		8.0				82.4		3.3				72.8		11.1
Leucomycin A5	y=310.19x+1.20	0.999	2-160	2.9	11.6	91.0	4.1				82.4		1.8				86.0		2.2				84.1		3.6				91.3		1.9				89.1		3.4
Oxolinic acid	y=3435.39x+2147.32	0.999	2-160	2.8	11.2	95.9	6.1				94.0		2.7				98.0		3.8				92.5		16.5				129		1.4				127		1.9
Flumequine	y=895.64x+177.80	0.998	2-160	2.3	9.2	104	5.4				98.6		1.0				102		3.5				89.7		16.9				126		2.6				126		2.3
Cloxacillin	y=264.77x-265.29	0.999	2-160	2.7	10.8	61.2	7.2				88.0		5.4				81.1		2.8				106		9.1				72.8		5.3				72.4		4.3
Josamycin	y=550.19x+337.48	0.998	2-160	2.8	11.2	64.9	7.2				76.8		2.8				85.6		3.0				76.6		3.1				92.4		1.2				94.4		2.2
Dicloxacillin	y=157.93x-38.90	0.998	2-160	3.2	12.8	84.3	9.1				73.7		5.9				76.5		2.3				110		9.9				74.0		7.5				73.6		2.0

*y*: peak area; *x*: mass concentration, μg/L.

#### 2.4.2 加标回收率和精密度

为考察方法的回收率和精密度,在某市地表水和生活污水(污水处理厂进口水)中添加低、中、高3个水平的混合标准溶液,使地表水中3个加标水平分别为25、100、250 ng/L,生活污水中3个加标水平分别为100、250、500 ng/L,每个水平做6个平行样。结果如表2所示,当水样为地表水时,22种目标化合物在低、中、高3个水平的回收率分别为61.2%~157%、73.1%~122%、76.5%~127%,相对标准偏差(RSD)分别为1.4%~11.4%、1.0%~20.1%、1.6%~21.9%,其中只有9%的目标化合物的回收率超过130%, 77.3%的目标化合物的回收率为70%~130%。当水样为生活污水时,22种目标化合物在低、中、高3个水平的回收率分别50.1%~128%、68.0%~129%、66.6%~127%, RSD分别为1.9%~16.9%、1.2%~11.6%、1.5%~11.1%,其中13.6%目标化合物的回收率小于70%。

#### 2.4.3 方法比较

从抗生素的类别、数量、富集水样体积、检出限、回收率、精密度和分析时间与其他采用固相萃取-液相色谱-串联质谱法同时测定环境水样中多种抗生素的文献方法进行比较,结果见附表1(详见https://www.chrom-China.com)。

结果表明,本文所建立方法的MDL和回收率等能够满足检测水样中多种抗生素的定量分析要求。此外,本方法富集水样体积少,仪器分析时间短,可有效缩短样品分析时间,达到快速检测的目的,为突发环境污染应急监测提供快速、高效、灵敏的分析方法依据。

### 2.5 实际水样分析

分别采集水库水、污水处理厂出口废水和畜禽养殖场废水各1瓶水样,同时采集某流域10个点位的地表水各1瓶,利用本研究建立的SPE-HPLC-MS/MS方法对水样进行检测,各水样中抗生素的含量见附表2。

从表中可看出在水库水样中未检出抗生素;污水处理厂总排口废水中仅林可霉素、克林霉素、氧氟沙星和诺氟沙星有检出,其余未检出;某流域10个地表水检出不同含量水平抗生素,其中林可霉素检出率为90%,克林霉素、竹桃霉素和交沙霉素检出率超过50%, 10个地表水样品中22种抗生素的含量范围为<LOQ~162 ng/L;畜禽养殖废水中大部分抗生素有检出,氧氟沙星的检出含量最高,为127 ng/L。

## 3 结论

采用固相萃取-高效液相色谱-串联质谱技术,通过重点优化固相萃取柱、水样pH值、水样中Na_2_EDTA加入量,以及目标化合物的色谱条件和质谱参数,建立了同时测定环境水样中22种抗生素的方法,方法成功用于水库、地表水、污水处理厂出口和畜禽养殖场等水样中抗生素的同时测定。该方法具有富集水样体积小、适用范围广、分析时间短、检出限低和回收率高等优势,为抗生素残留的标准制定和实际环境监测,尤其为突发环境污染应急监测提供补充和参考,同时为摸清新污染物环境赋存底数和新污染物治理与管控提供有力支撑。
